# Risk of Periodontal Diseases in Women With Polycystic Ovary Syndrome: An Overview

**DOI:** 10.7759/cureus.47169

**Published:** 2023-10-17

**Authors:** Netal Rathi, Amit Reche

**Affiliations:** 1 Public Health Dentistry, Sharad Pawar Dental College, Datta Meghe Institute of Higher Education and Research, Wardha, IND

**Keywords:** hormonal imbalance, obesity, insulin resistance, periodontal diseases, polycystic ovary syndrome

## Abstract

Polycystic ovary syndrome (PCOS) is the most prevalent condition seen in reproductive-aged women, which has a negative impact on their health system. There is a serious concern for women having PCOS that they may experience long-term metabolic conditions. PCOS also has a negative impact on periodontium components such as gingiva, periodontal ligament (PDL), and alveolar bone. It has been said that there may be a bidirectional link between PCOS and periodontal diseases. Moreover, PCOS and periodontal disorders both have common risk factors. Periodontal diseases are exacerbated by systemic low-grade inflammation, including obesity, constant immunological imbalance, and oxidative stress caused by PCOS. On the other hand, periodontal diseases can also increase the risk of causing PCOS. According to recent data, women with PCOS may be more likely to suffer from periodontal diseases. A PubMed and Google Scholar search was conducted for literature relating to PCOS and its relationship with different comorbidities which also included periodontal disorders. Analyses were performed, and data was synthesized and assembled in a presentable form. Therefore, the focus of this review will be on the relationship between PCOS and periodontal disorders, as well as the risk factors for both. However, in order to establish a more distinct and solid link, more studies with a large sample size need to be done.

## Introduction and background

Polycystic ovary syndrome (PCOS) is a common endocrine system disorder in women primarily affecting the reproductive system [1]. It affects at least 8%-13% of reproductive females [2]. According to WHO, 70% of the cases remain undiagnosed. Patients suffering from PCOS exhibit various symptoms related to menstrual dysfunction and excess androgens, all of which have a negative influence on their lifestyle. The patients can be at higher risk for multiple comorbidities, which include resistance to insulin, type II diabetes mellitus, cardiovascular diseases (CVD), obesity, infertility, malignancy, and psychosocial disorders [3]. PCOS is often linked with low-grade chronic systemic inflammation [4]. Certain proinflammatory cytokines have been observed to be raised in women with PCOS.

Periodontal diseases including periodontitis and gingivitis are inflammatory conditions that elicit innate, adaptive, and inflammatory immunological responses. They are brought on by pathogenic microorganisms present in the oral microflora [5]. Periodontal disorders have been linked with an altered vascular response, increased levels of adhesion molecules (for example vascular cell adhesion molecule-1 and intercellular adhesion molecule-1) and higher expression of local and systemic inflammatory cytokines, including tumour necrosis factor-α (TNF-α), interleukin (IL)-1β, IL-6 and monocyte chemoattractant protein, all of which impair endothelial function [6-8]. Periodontal disorders can cause tooth loss, affecting one's capability to maintain a healthy lifestyle, and can also affect speech and social behaviour [9].

## Review

Search methodology

**Figure 1:**
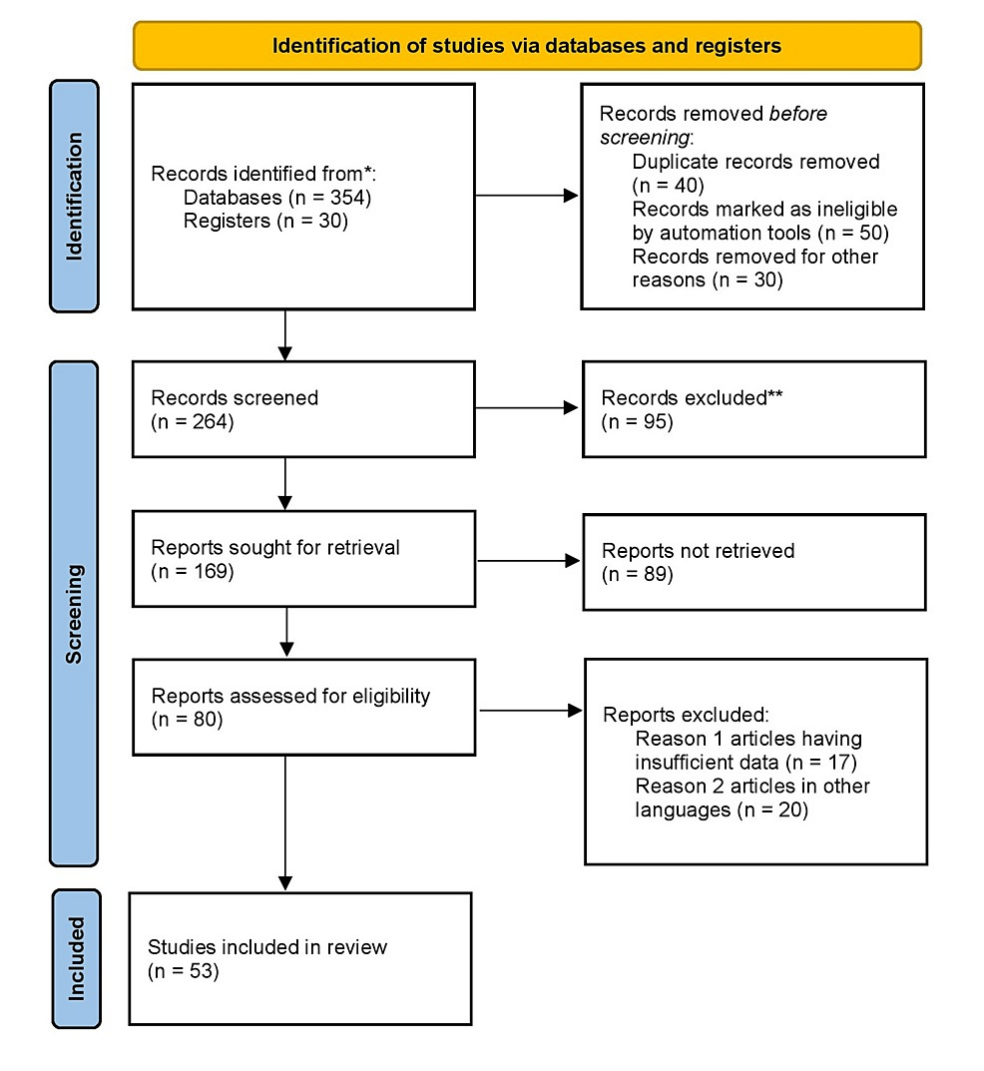
PRISMA flow diagram of search strategy PRISMA: Preferred Reporting Items for Systematic Reviews and Meta-Analyses

A comprehensive PubMed and Google Scholar search was carried out using keywords such as “Polycystic ovary syndrome”, “Hyperandrogenism”, “Insulin resistance”, “Periodontal diseases”, and “Hormonal imbalance”, “stress” to discover papers published up to September 2022. After reading all of the relevant articles, analysis was performed, and data was organized in a sequential manner to offer a concise overview of PCOS and its relationship to other different comorbidities, as well as to investigate in depth the possible relationship between PCOS and periodontal diseases. For inclusion, studies in English, both published and unpublished, were considered. Because of the lack of resources and the reviewer's inability to acquire full-text articles, we excluded research published in other languages. Figure 1 shows the Preferred Reporting Items for Systematic Reviews and Meta-Analyses (PRISMA) flow chart for the selection strategy of the article.

Polycystic ovary syndrome (PCOS)

PCOS is a hormonal condition distinguished by features such as oligomenorrhea (irregular and inconsistent menstruation) or amenorrhea (absence of menstruation), oligo-ovulation (infrequent ovulation), hyperandrogenism (hirsutism, acne, or androgenic alopecia). It was first documented by Dr. Stein and Dr. Levanthal in 1935 [10]. It affects many women of reproductive age. High levels of testosterone, insulin resistance (IR), and enlarged, dysfunctional ovaries are frequently associated with this disease [11]. Table 1 shows the characteristics and causes of PCOS.

**Table 1:** Characteristics and causes of PCOS PCOS: Polycystic ovary syndrome

CHARACTERISTICS	CAUSES
Insulin resistance	Hormonal imbalance
Hyperandrogenism	Genetic predisposition
Obesity	Stimulation in adrenals during childhood
Irregular menstrual cycle	Elevated insulin levels
Infertility	Contraceptive pills
Insulin resistance	Low-grade inflammation

Pathophysiology of PCOS

**Figure 2:**
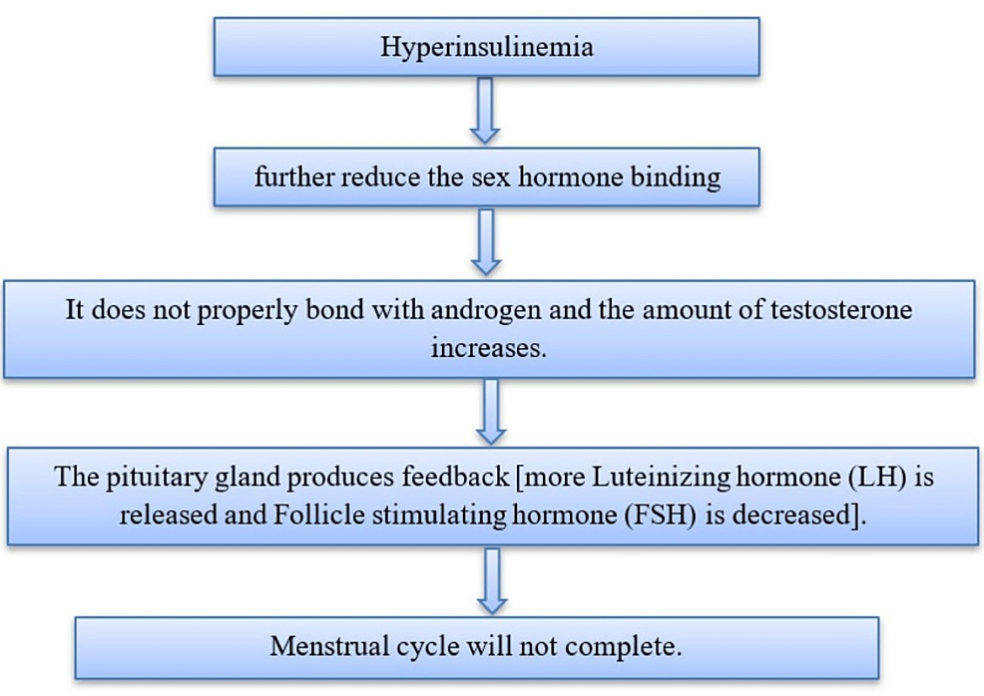
Pathophysiology of PCOS PCOS: Polycystic ovary syndrome Image credits: Netal Rathi

**Figure 3:**
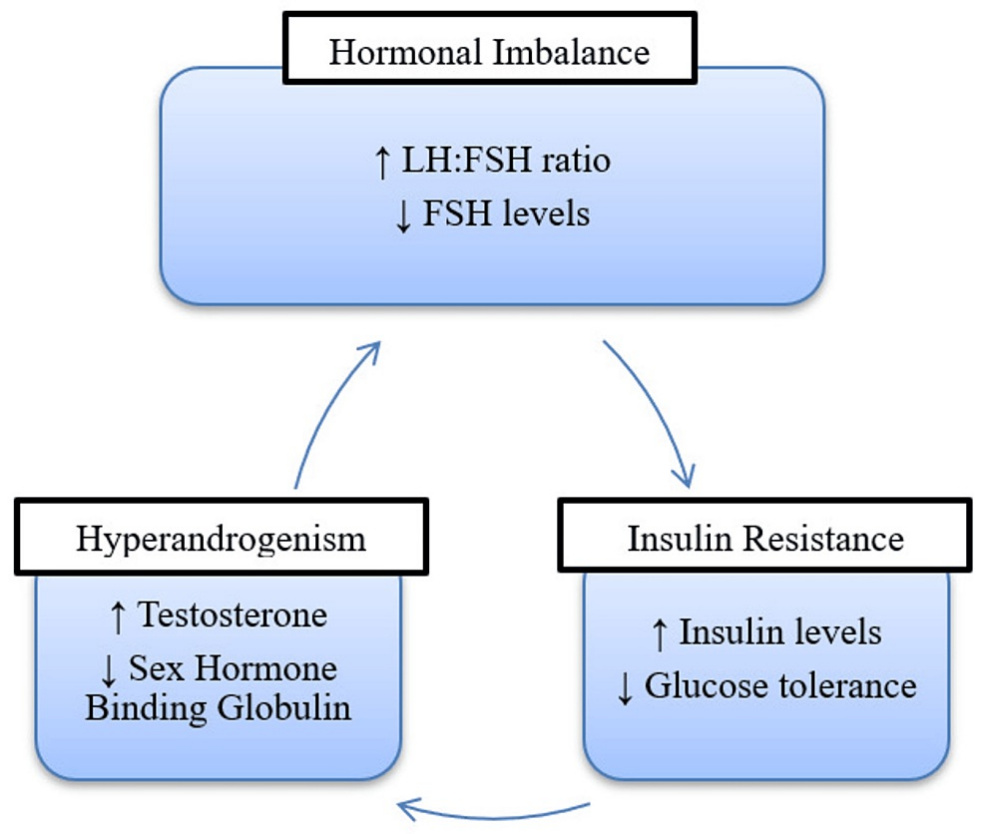
Changes occurring in PCOS LH:  Luteinizing hormone; FSH: Follicle-stimulating hormone Image credits: Netal Rathi

Normally, insulin in the body takes up glucose from carbohydrate-rich food and utilizes it. In PCOS, insulin resistance (IR) occurs resulting in no glucose uptake leading to the pancreas producing more insulin, this condition is known as hyperinsulinemia. Figure 2 shows the pathophysiology of PCOS and Figure 3 indicates changes occurring in PCOS.

Diagnosis

Clinical symptom-based classifications were created. The National Institutes of Health (NIH) Criteria given in 1990, the Rotterdam's Criteria in 2003, the Androgen Excess PCOS Criteria in 2006, and the NIH extension of American Society for Reproductive Medicine (2003) criteria provided in 2012/International PCOS guidelines given in 2018 were among these categories. Table 2 shows the classification for diagnosis of PCOS.

**Table 2:** Classification for diagnosis of PCOS PCOS: Polycystic ovary syndrome

National Institutes of Health (1990)	Rotterdam Criteria (2003)	Androgen Excess Criteria (2006)	NIH 2012/ International PCOS Guidelines 2018
Ovulatory Dysfunction (Chronic anovulation)	Hyperandrogenism	Hyperandrogenism	Hyperandrogenism (HA)
	Ovulatory Dysfunction (Oligo/anovulation)	Ovulatory Dysfunction (Oligo/anovulation)	Ovulatory Dysfunction (OD)(Oligo/anovulation)
Hyperandrogenism	Polycystic ovarian morphology	-	Polycystic ovarian morphology (PCOM)
Both criteria are needed.	Two of the three criteria are needed.	Both criteria are needed.	Two of the three criteria and phenotype identification are needed. Phenotype identification- HA+OD HA+OD+PCOM HA+PCOM OD+PCOM

Factors linking PCOS and periodontal diseases

Oral and Intestinal Microflora

Periodontitis is mainly a biofilm-induced disease that begins and progresses as a result of interactions among the host and the oral microflora. The movement of immune cells from the sulcus of the gingiva to the oral ecosystem may be accelerated by a number of conditions, including inflammatory cytokines, toxins, and hormone instability [12]. Hormonal changes occurring in PCOS alter the amount of possible periodontal microbes in saliva and the body’s immune responses in the presence of gingivitis. This can be shown as an increase in the amount of progesterone and estrogen in periodontal tissues, which provide the nutrients that are required for the growth of microorganisms [13].

*Fusobacterium nucleatum* (Fn), an anaerobic bacteria in supra and subgingival biofilms along with *Porphyromonas gingivalis* (Pg), *Streptococcus gordonii *(Sg), and *Treponema denticola* (Td) accelerate biofilm formation. According to Akcali et al. [14], patients who suffer from PCOS and gingivitis had higher salivary levels of Pg and Fn when compared to healthy controls and patients having PCOS but no gingivitis. It was also found that the number of Pg had the highest correlation with the severity of periodontal disease among the red-complex bacteria [15]. Women suffering from PCOS had increased levels of Fusobacterium and decreased levels of Actinobacteria in their salivary microbiomes [16]. Additionally, patients with PCOS as a comorbid condition had higher abundances of *Tannerella forsythia* (Tf) in their sulcular fluid [14]. Sex hormones can also play a role in altering oral microflora as higher amounts of Fn species were seen in women than in men [17].

*Bacteroides vulgatus* was found in high abundance in gut microflora of PCOS patients [18]. In periodontal conditions, disruption of the salivary microflora results in dysbiosis of the gut flora. Salivary microorganisms from periodontitis survive in the intestine and cause dysbiosis of the intestinal microbiota. The concentration of Fn and Porphyromonadaceae in mice having severe periodontal disease characterized the change in composition of the gut microbiome [19]. Therefore, periodontitis may enhance a person's risk of developing PCOS by altering the host metabolism, intestinal permeability, and intestinal flora.

Inflammation

Inflammation is a key factor in the pathophysiology of both PCOS and periodontal disorders. Low-grade chronic inflammation, a characteristic hallmark of PCOS, is expected to play a substantial role in the development of periodontal disorders. Numerous pieces of research have shown that inflammatory cytokines like tumor necrosis factor alpha (TNF-α), C-reactive protein (CRP), and interleukin-6 play a role in the connection between PCOS and periodontal disorders. CRP which is considered one of the essential markers of inflammation is seen raised in many diseases including PCOS [20]. Periodontitis patients exhibit increased blood CRP levels as well as proinflammatory cytokines such as TNF-α and interleukin-1 in their serum and gingival crevicular fluid (GCF). In long-term infections such as periodontitis, high serum CRP levels and other proinflammatory circumstances may promote systemic inflammation and oxidative stress, leading to IR, which are features of PCOS. Patients with periodontitis have higher levels of inflammatory biomarkers such as CRP and interleukin-6 in both gingiva and serum [21]. According to Nicklas et al. [22], long-term lifestyle adjustments in PCOS patients, such as lower levels of CRP, Interleukin-6, and TNF-α levels, reduce the systemic inflammatory burden and, as a result, reduces periodontitis. Rahiminejad et al. discovered that nonobese women having PCOS had a greater prevalence of periodontal disease than systemically healthy people, showing that systemic inflammation plays a role [23].

Another such marker of low-grade inflammation is the WBC. In a case-control study, Orio et al. [24] discovered that women suffering from PCOS had an increased leukocyte count than systemically healthy controls. Similar to this, people with persistent periodontitis may experience an elevated white blood cell count [25].

Obesity

According to some studies, obesity affects 30-70% of women suffering from PCOS [26]. Furthermore, research demonstrates that PCOS women have more abdominal fat than weight-matched controls, which causes hyperinsulinemia and IR [27]. Adipose tissues release cytokines and inflammatory mediators that further regulate inflammation. PCOS was linked to increased levels of CRP, interleukin-6, and TNF- α, all of which contribute to chronic low-grade inflammation and raise the risk of periodontal disease [28].

Oxidative Stress (OS)

It is said that PCOS triggers OS. The OS induced by PCOS has an effect on gingival inflammation. Furthermore, indicators of oxidative stress were discovered in the peripheral blood of patients with chronic periodontal disorder and PCOS [29]. A meta-analysis [30] found that patients with periodontitis and PCOS had higher levels of OS. Compared to women with PCOS alone, women with periodontal disease had lower total antioxidant levels [31].

Bone Resorption

Women having PCOS are more likely to develop osteoporosis [32]. Numerous data available have discovered connections between periodontal diseases such as increased tooth loss, alveolar bone loss, clinical attachment loss, and low bone mineral density (BMD) [33]. Hyperandrogenemia, obesity, and IR are considered to contribute to a higher BMD in women having PCOS [34]. In these women, IR decreases the osteoprotegerin (OPG) expression and increases the expression of RANKL, both of which are important for the resorption of bone [35]. These results suggest that hormonal imbalance in PCOS-affected women may accelerate bone resorption and contribute to periodontal disorders.

Hormonal Imbalance

As PCOS is a hormonal condition, the association seen between PCOS and Periodontal diseases coincides with the hypothesis that female sex hormones can lead to gingivitis [36]. It also plays an important role in periodontal healing [37]. Patients with PCOS may experience estrogen deficiency, which lowers BMD [38]. It has been proposed that estrogen insufficiency, which raises women's risk for osteoporosis, may also affect the hard and soft tissues of the oral cavity, making them more vulnerable to damage from periodontal disease [39]. Estrogen shortage also results in lower calcium absorption and poor protection against bone resorption, both of which may lead to systemic bone loss.

Risk Factors

Multiple widespread risk factors for periodontal diseases may also be seen in women with PCOS. Table 3 concludes the risk factors for PCOS and periodontal diseases.

**Table 3:** Risk factors for both PCOS and periodontal diseases PCOS: Polycystic ovary syndrome

Risks for PCOS	Risks for Periodontal diseases
Insulin resistance Type II diabetes	Age
High blood pressure	Genetic
	Obesity
Cardiac diseases	Smoking
Endometrial Cancer	Tobacco consumption
Obesity	Certain medications
	Type II diabetes
Osteoporosis	Stress
Miscarriage	Low calcium and Vitamin D intake
	Cardiac diseases

Association of PCOS with other conditions

According to Lim et al. [40], obesity can aggravate preexisting clinical, hormonal, and metabolic conditions in women suffering from PCOS. It was concluded by Nicandri and Hoeger [41] that obesity affects more than 60% of people with PCOS. IR is common in PCOS women, ranging from 50% to 70% [42]. According to Legro et al. [43], the occurrence of defective glucose tolerance and type II diabetes mellitus in females suffering from PCOS is 30%-40% and 7.5%-10%, respectively.

Females having PCOS have more tendency to develop CVDs, particularly high blood pressure, dyslipidemia, and high levels of insulin, at a younger age when compared with women not suffering from PCOS [44]. According to a study done by Wild et al. [45], PCOS women may be highly prone to developing hypertension. PCOS women are also at risk of having severe coronary artery disease [46]. Recent data suggests that PCOS women regardless of their age have a higher chance of developing endometrial cancer [47]. Some research also shows that women having PCOS have lower BMD than healthy controls [48].

Studies related to the current review

Table 4 shows various studies performed which show a positive link between PCOS and periodontal diseases [49-53]. All the studies were done on women aged 15 to 45 years. The criteria commonly used for diagnosis was the Rotterdam criteria.

**Table 4:** Studies linking PCOS and periodontal diseases PCOS: Polycystic ovary syndrome; IL: Interleukin; TNF: Tumor necrosis factor; CAL: Clinical attachment level; PI: Plaque index; BOP: Bleeding on probing

Sr. no.	Author	Study	Finding
1.	Dursun et al. [49]	reported an association between PCOS and periodontal diseases for the first time.	increased susceptibility for periodontal disease in PCOS
2.	Ozcaka et al. [50]	examined the levels of proinflammatory cytokines in gingival crevicular fluid (GCF), saliva, and serum in patients with and without polycystic ovary syndrome (PCOS).	PCOS and gingival inflammation act synergistically, thereby increasing the levels of IL-6 and TNF-α.
3.	Akcali et al. [51]	analyzed the levels of matrix metalloproteinase-8 (MMP-8) and tissue inhibitors of MMP-1 (TIMP-1) in saliva and serum samples from women with polycystic ovarian syndrome who have varied degrees of gingival inflammation.	increased levels of matrix metalloproteinase-8 (MMP-8) in whole saliva and serum in patients with periodontal diseases and PCOS compared with healthy controls.
4.	Kellesarian et al.[52]	association between periodontal disease and polycystic ovary syndrome	women with PCOS are at increased risk of periodontal disease
5.	Rahiminejad et al. [53]	prevalence of periodontal disease in women with polycystic ovary syndrome and healthy controls	higher CAL, PI and BOP in patients with PCOS compared with healthy controls.

## Conclusions

The above data demonstrates a positive relationship between PCOS and periodontal disorders. Women with PCOS are at a higher risk of suffering from periodontal problems which can compromise their lifestyle. Evidence showed a bidirectional link between the two. Periodontal disease can also produce persistent subclinical inflammation that results in IR and the onset of type 2 diabetes, which is a common symptom of PCOS. Moreover, both diseases also share common risk factors which support the association. Early diagnosis for any periodontal disease is very crucial. It can help to prevent further complications leading to healthy periodontal health. Therefore, healthcare professionals would need to encourage women diagnosed with PCOS to maintain proper oral hygiene and encourage them to see a dentist on a frequent basis in order to avoid future periodontal risk.
